# Genotyping *Brucella canis* isolates using a highly discriminatory multilocus variable-number tandem-repeat analysis (MLVA) assay

**DOI:** 10.1038/s41598-017-01114-7

**Published:** 2017-04-21

**Authors:** Yi Yang, Yin Wang, Elizabeth Poulsen, Russell Ransburgh, Xuming Liu, Baoyan An, Nanyan Lu, Gary Anderson, Chengming Wang, Jianfa Bai

**Affiliations:** 1 Kansas State Veterinary Diagnostic Laboratory, College of Veterinary Medicine, Kansas State University, Manhattan, KS, 66502 USA; 2 Jiangsu Co-Innovation Center for the Prevention and Control of Important Animal Infectious Diseases and Zoonoses, Yangzhou University College of Veterinary Medicine, Yangzhou, Jiangsu China; 3 Department of Diagnostic Medicine/Pathobiology, College of Veterinary Medicine, Kansas State University, Manhattan, KS 66502 USA; 4 Division of Biology, Kansas State University, Manhattan, KS 66506 USA; 5 Department of Pathobiology, College of Veterinary Medicine, Auburn University, Auburn, AL 36849 USA

## Abstract

Differentiation of *Brucella canis* from other *Brucella* species are mainly performed through PCR-based methods and multilocus variable-number tandem-repeat (VNTR) analysis (MLVA) procedures. Both PCR-based and MLVA methods are limited in discriminating *B*. *canis* strains. A new MLVA-13Bc method for *B*. *canis* genotyping was established by combining eight newly-developed VNTRs with five published ones. During 2010 and 2016, 377 *B*. *canis* PCR-positives were identified from 6,844 canine blood samples from 22 U.S. states, resulting in 229 *B*. *canis* isolates. The MLVA-13Bc method was able to differentiate each of these 229 isolates. The Hunter-Gaston Discriminatory Index of the individual VNTR loci ranged from 0.516 to 0.934 and the combined discriminatory index reached 1.000. Three major clusters (A, B and C) and 10 genotype groups were identified from the 229 *B*. *canis* isolates. Cluster A mainly contains genotype groups 1 and 2, and a few group 3 isolates; nearly all Cluster B isolates were from group 6; other genotype groups were classified into Cluster C. Our newly developed MLVA-13Bc assay is a highly discriminatory assay for *B*. *canis* genotyping, and can serve as a useful molecular epidemiological tool, especially for tracing the source of contamination in an event of a *B*. *canis* outbreak.

## Introduction

*Brucella canis* is a gram-negative, facultative intracellular pathogen mainly responsible for causing canine brucellosis. *B*. *canis* infections can lead to abortion in females and epididymitis and prostatitis in males^1^. Main routes of transmission are through direct contact with aborted fetus, placenta, fetal fluids, or vaginal discharge^2^. The organism stays in the animal system longer than other *Brucella* spp., making intervention more challenging^3^. An aborting bitch can discharge the bacterium at very high concentrations for 4–6 weeks after a single abortion^2, 4^. The disease has been widely reported in different continents^2, 5–8^, and it has been a major problem in canine breeding facilities^1^. Infection rate of canine brucellosis is on the rise. The Wisconsin Veterinary Diagnostic Laboratory tested 510 samples in 1995 and 1996, and only resulted in 10 positives (1.96%); Testing on 174 samples during 2003 and 2004 by the same lab resulted in 4.6% positivity^9^. The positive rate can reach up to 26.8% during an outbreak^9^. Although it is uncommon, human *B*. *canis* infections have been reported^10^ with symptoms from mild flu-like illnesses to more severe complications^11–14^. Thus, its zoonotic potential has become a public health concern.

Variable number of tandem repeat (VNTR) and multilocus VNTR analysis (MLVA) have been widely used for genotyping strains of different bacterial species^15–20^. Several MLVA systems have been developed and described for the genotyping of *Brucella* species and biovars^21–23^. Bricker *et al*.^21^ used a MLVA method named HOOF-Prints with eight tandem repeat (TR) loci (MLVA-8) and was able to differentiate *Brucella* isolates at both the species and biovar levels. A rather comprehensive screening of 107 TRs identified 15 TRs (MLVA-15) that were more informative and were able to differentiate most *Brucella* species and for some strains even at the biovar level^22^. Al Dahouk *et al*.^24^ added another TR into the MLVA-15 method to form a new assay called MLVA-16 and was used to study genetic diversity of 128 human *B*. *melitensis* strains. The study identified 110 genotypes that differentiated most of the 128 strains, yet the MLVA-16 method provided much lower discriminatory power against the eight *B*. *canis* and 18 *B*. *ovis* strains in the study. Using MLVA-16, *B*. *suis* biovar 1, *B*. *suis* biovar 2, *B*. *abortus*, *B*. *melitensis* and *B*. *ovis* can be clearly identified. *B*. *suis* biovar 5, *B*. *neotomae* and the marine mammal strains are closely related strains, and they can be differentiated by this method. However, this method appeared to be insufficient in differentiating strains within the species of *B*. *canis* in different studies, especially for those that were from closely related geographic regions^22, 24, 25^. Whatmore *et al*.^23^ grouped 121 *Brucella* isolates into 119 genotypes based on 21 VNTR loci. The approach based on 21 VNTR loci provided better strain genotyping information for *B*. *abortus, B*. *melitensis* and *B*. *suis*, but was less informative in differentiating strains of *B*. *canis, B*. *ovis* and *B*. *neotomae*. Therefore, the goal of this study were: 1) to develop and validate a MLVA genotyping method for the differentiation of *B*. *canis* strains; and 2) to study genetic diversity of a collection of 229 *B*. *canis* isolates collected from the US in recent years using the newly developed MLVA-13Bc method.

## Results

### PCR identification and isolation of *B*. *canis* strains from canine blood samples

From a total of 6,844 canine blood samples, the duplex diagnostic PCR identified 377 *Brucella* positives, and all *Brucella* positives were confirmed to be *B*. *canis* strains. The MLVA-13Bc PCR amplifications for 10 genotype groups visualized by QIAxcel were shown in Figure S1. Among the 377 PCR-positive samples, 229 *B*. *canis* isolates were obtained. Selected positive samples (n = 20) were further verified by sequencing a region flanking a 976 bp fragment that is deleted only from the *B*. *canis* genome, and is intact in all other *Brucella* species^26, 27^. Positive samples were observed in 10 states, mainly from the Midwest region, of the US with an average positive rate of 5.5% (377/6,844). The positive rate for the 6,844 samples ranged from 3.8% (58/1,533 IN) to 23.1% (3/13 MS) when sorted by sampling state, and varied from 2.9% (13/445 2015) to 9.1% (24/264 2013) if sorted by sampling year. The remaining 99 samples collected from 12 other states were negative for *B*. *canis* (Table 1).

**Table 1:** Prevalence of *Brucella canis* in canine populations collected from 22 states of the US.

State	2010	2011	2012	2013	2014	2015	2016	Total
CO	14/99							14/99 (14.1%)^a^
IA				8/192	23/133	1/56	3/42	35/423 (8.3%)
ID						4/8	5/43	9/51 (17.6%)
IN			11/132	31/878	10/280	3/169	3/74	58/1,533 (3.8%)
KS	4/103	5/166	1/12	37/499	7/120	0/76	1/60	55/1,036 (5.3%)
MN				7/86	3/25	0/2		10/113 (8.8%)
MO		4/5	4/75	42/677	6/143	5/106	3/34	64/1,040 (6.2%)
MS							3/13	3/13 (23.1%)
OH			8/44	38/984	1/37	0/4		47/1,069 (4.4%)
OK	49/824	29/512		1/13	3/17		0/2	82/1,368 (6.0%)
FL					0/1	0/16	0/8	0/25 (0%)
IL				0/14		0/1		0/15 (0%)
ND						0/1		0/1 (0%)
NE			0/1		0/12	0/2		0/15 (0%)
NM						0/1		0/1 (0%)
NY				0/1		0/1		0/2 (0%)
PA				0/2				0/2 (0%)
SD				0/4	0/11		0/4	0/19 (0%)
TN					0/2			0/2 (0%)
TX					0/10			0/10 (0%)
VA				0/1	0/1		0/1	0/3 (0%)
WI					0/2	0/2		0/4 (0%)
Total	67/1,026 (6.5%)	38/683 (5.6%)	24/264 (9.1%)	164/3,351 (4.9%)	53/794 (6.7%)	13/445 (2.9%)	18/281 (6.4%)	377/6,844 (5.5%)
Isolates^**b**^	28^c^	32	16	130	0^d^	7^c^	16	229

^a^Positive/total (positive rate%).

^b^Number of *B*. *canis* isolates obtained from positive samples.

^c^Not all positive samples were subjected to bacterial isolation for 2010 and 2015.

^d^No sample was subjected to bacterial isolation for year 2014.

### Development and optimization of the MLVA genotyping method for *B*. *canis*

We particularly analyzed the polymorphism generated by each of the 16 VNTR loci in the MLVA-16 method^22, 24^ using published data. Our analysis indicated that five of the 16 loci published earlier should generate genetic polymorphism among different *B*. *canis* strains (Table 2), yet may not be enough to differentiate the 229 isolates in our collection. Additional 8 loci (starts with “BCTR”) that can potentially aid to differentiate strains within the *B*. *canis* species were selected for the design of new VNTRs. Results of the 13 individual VNTRs on 20 isolates generated large genetic polymorphism, indicating they are useful loci for genotyping strains of *B*. *canis* (Figure S2). Based on the average size range and primer annealing temperatures of each VNTR locus, 12 of them were grouped into 6 duplex PCR reactions, namely Reaction 1 (for loci BCTR09 and BCTR06), 2 (BCTR12 and Bruce07), 3 (BCTR03 and Bruce16), 4 (BCTR02 and Bruce09), 5 (BCTR01 and BCTR08), and 6 (BCTR11 and Bruce04), respectively. Reaction 7 is a singular one for locus Bruce18 (Table 2). All primers were specific to targeted regions and did not amplify other regions of the *B*. *canis* genome. The results were compared to individual PCR reactions. No interference was observed while comparing the singular and duplex PCR reactions. There were no overlapping amplicons observed between the 2 loci within each duplex reaction. The result of Sanger sequencing (data not shown) was consistent with the size obtained by the QIAxcel Advanced System.

**Table 2:** Primer and VNTR loci information for the MLVA-13Bc genotyping method developed in this study.

Reaction^a^	VNTR locus^b^	CHR^c^	Forward Primer	Reverse Primer	Annealing Temperature	Repeat Length (bp)	No. of Repeat	Amplicon Length (bp)	Location on Genome^c^	Reference
1	BCTR06	I	CCTTGACTGAAAAAGGGTGATT	AGAAGGCACTCGATCTCATCG	58 °C	8	3–15	119–215	734705–734855	This study
	BCTR09	II	GCTTTATCTTATCTGATTCTTTCAAATTC	GCTGACAGAACCCATCGTCAAT	58 °C	8	9–25	241–369	73060–73316	This study
2	Bruce07	I	GCTGACGGGGAAGAACATCTAT	ACCCTTTTTCAGTCAAGGCAAA	58 °C	8	1–7	142–190	734557–734722	22
	BCTR12	II	GATCCTGATCGTTCGCTTCG	CGTCGATGCACGGACTATCG	58 °C	8	4–17	402–506	975304–975737	This study
3	BCTR03	I	AAGCGGCGAGAGTTTGTCGT	GATGAAGTCGTTGCTGCCTATTAC	58 °C	8	5–20	409–529	86891–87307	This study
	Bruce16	II	ACGGGAGTTTTTGTTGCTCAAT	GGCCATGTTTCCGTTGATTTAT	58 °C	8	1–12	144–232	748957–749140	22
4	BCTR02	I	GGAACCCGACAGTGAACACG	ATAATCTCGACACGCAGGCAAC	60 °C	8	1–23	346–522	64479–64896	This study
	Bruce09	I	GCGGATTCGTTCTTCAGTTATC	GGGAGTATGTTTTGGTTGTACATAG	60 °C	8	2–16	124–236	1398399–1398578	22
5	BCTR01	I	GTTTTTGGTTGCGCATGG	GGTGGATTTCAGGAACAACG	62 °C	8	8–24	214–342	62690–62943	This study
	BCTR08	II	GTCTGCTGCACTCAAGACGATC	GAATTTGAAAGAATCAGATAAGATAAAGC	62 °C	8	2–10	182–246	72899–73088	This study
6	Bruce04	I	CTGACGAAGGGAAGGCAATAAG	CGATCTGGAGATTATCGGGAAG	62 °C	8	2–7	160–200	438775–438966	22
	BCTR11	II	CAGAATTTTCGAGGCATTCG	GAGACGGTGGGGGTTAAAG	62 °C	8	3–13	195–275	969122–969364	This study
7	Bruce18	II	TATGTTAGGGCAATAGGGCAGT	GATGGTTGAGAGCATTGTGAAG	60 °C	8	2–6	138–170	958389–958534	22

^a^The primers were grouped into 6 duplex PCR reactions plus a singular PCR reaction.

^b^VNTR: Variable number tandem repeat.

^c^Chromosome number to which the VNTR loci are located on the genome of *B*. *canis* strain ATCC 23365 (Chromosome I: NC_010103.1, Chromosome II: NC_010104.1).

### Genotyping 229 *B*. *canis* isolates using MLVA-13Bc method

The newly established method (Table 2) was then used for genotyping the remaining 229 isolates. Copy numbers of the tandem repeats for each locus were compiled to indicate the genotype for each isolate, and then used to further group the isolate into one of the 10 genotype groups (Figure S2). Each of the 229 isolates can be differentiated by this newly-developed MLVA-13Bc (MLVA-13 for *B*. *canis*) method. To evaluate the discriminatory power of the selected loci, the Hunter and Gaston^28^ discrimination index (HGDI) was calculated for the MLVA-13Bc method as a combined method, and for each of the 13 loci used in this study. Among these 229 isolates, the newly developed MLVA assay allowed the classification of all isolates to unequivocal genotypes (HGDI = 1.000). The HGDI value ranged from 0.516 to 0.934 among the 13 individual VNTR loci (Table 3).

**Table 3:** Number of alleles and discriminatory index generated by the MLVA-13Bc method and by individual VNTR locus on 229 *B*. *canis* isolates.

Locus	No. of alleles	HGDI^a^	CI 95%^b^
MLVA-13	229	1.000	1.000–1.000
BCTR06	16	0.898	0.886–0.911
BCTR09	20	0.918	0.908–0.928
Bruce07	7	0.502	0.434–0.570
BCTR12	13	0.881	0.868–0.895
BCTR03	16	0.891	0.875–0.907
Bruce16	11	0.817	0.785–0.849
BCTR02	22	0.934	0.925–0.943
Bruce09	14	0.881	0.865–0.897
BCTR01	17	0.884	0.867–0.900
BCTR08	10	0.858	0.844–0.872
Bruce04	10	0.773	0.750–0.796
BCTR11	13	0.907	0.900–0.914
Bruce18	4	0.516	0.452–0.581

^a^Hunter-Gaston Discriminatory Index (HGDI).

^b^95% confidence interval of HGDI for each locus.

**Figure 1:**
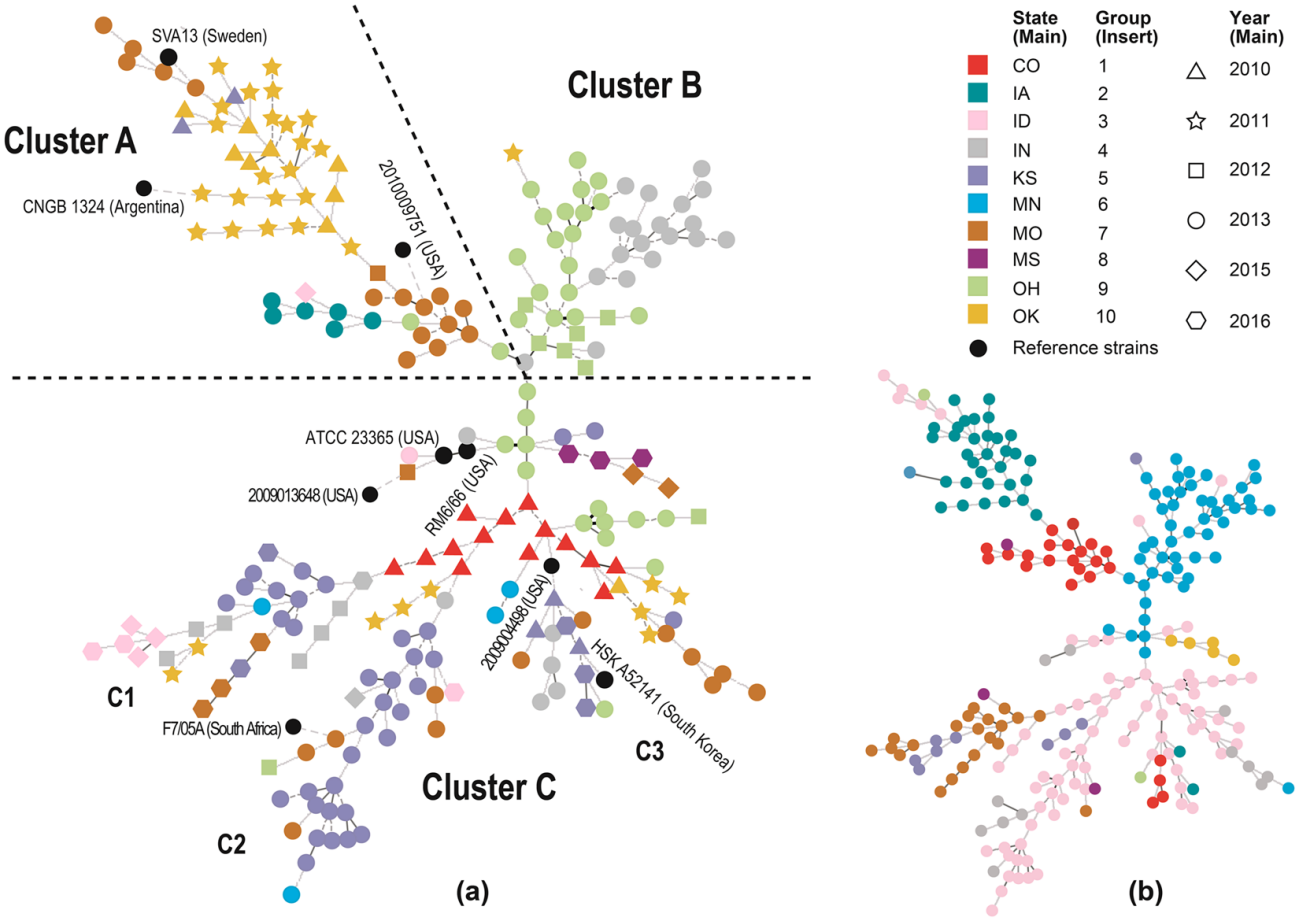
Geographical and temporal distribution of 229 *B*. *canis* isolates in 10 U.S. states. Different colors indicate different states in the main chart, or one of the 10 genotype groups of *B*. *canis* isolates in the inserted chart. Red: Colorado in the main chart, or group 1 in the insert chart; dark teal: Iowa or group 2; pink: Idaho or group 3; gray: Indiana or group 4; lavender: Kansas or group 5; light teal: Minnesota or group 6; orange: Missouri or group 7; maroon: Mississippi or group 8; light green: Ohio or group 9; and light orange: Oklahoma or group 10.

Genotyping of the 229 *B*. *canis* isolates by the newly developed MLVA-13Bc method identified 10 genotype groups (group 1 - group 10), with the similarity cutoff at 20% (Figure S2). The US strain 2010009751 was classified into group 1; the Korean strain HSK A52141 was in group 2; US strains ATCC 23365, RM6/66 and 2009004498 were classified into group 3; US strain 2009013648 and the South Africa strain F7/05 A were in group 4; the Argentina strain CNGB 1342 was in group 6; and the Sweden strain SVA13 was in group 9. Group 3 is the largest group with 76 isolates widely spread in 8 states, and it is the predominant group in Colorado (100%) and Kansas (71%). The second largest group, group 6, had strains only from Ohio (71%), Indiana (58%) and Missouri (3%). The 33 isolates in group 2 were mostly obtained from Oklahoma (30); only two from Kansas and one from Missouri. The 22 isolates in group 1 were from Iowa, Missouri, Indiana and Ohio, while the 21 isolates in group 7 were from Idaho, Kansas, Minnesota, Missouri and Indiana, which were widely spread in the US. There were no more than 10 isolates in groups 4, 5, 8, 9 and 10, and each of these groups was observed in only one or two states (Figure S2). Distribution of the 10 genotype groups were also illustrated in a cluster analysis (Insert in Fig. 1).

### Cluster analysis and geographical and temporal distributions of *B*. *canis* strains

**Figure 2:**
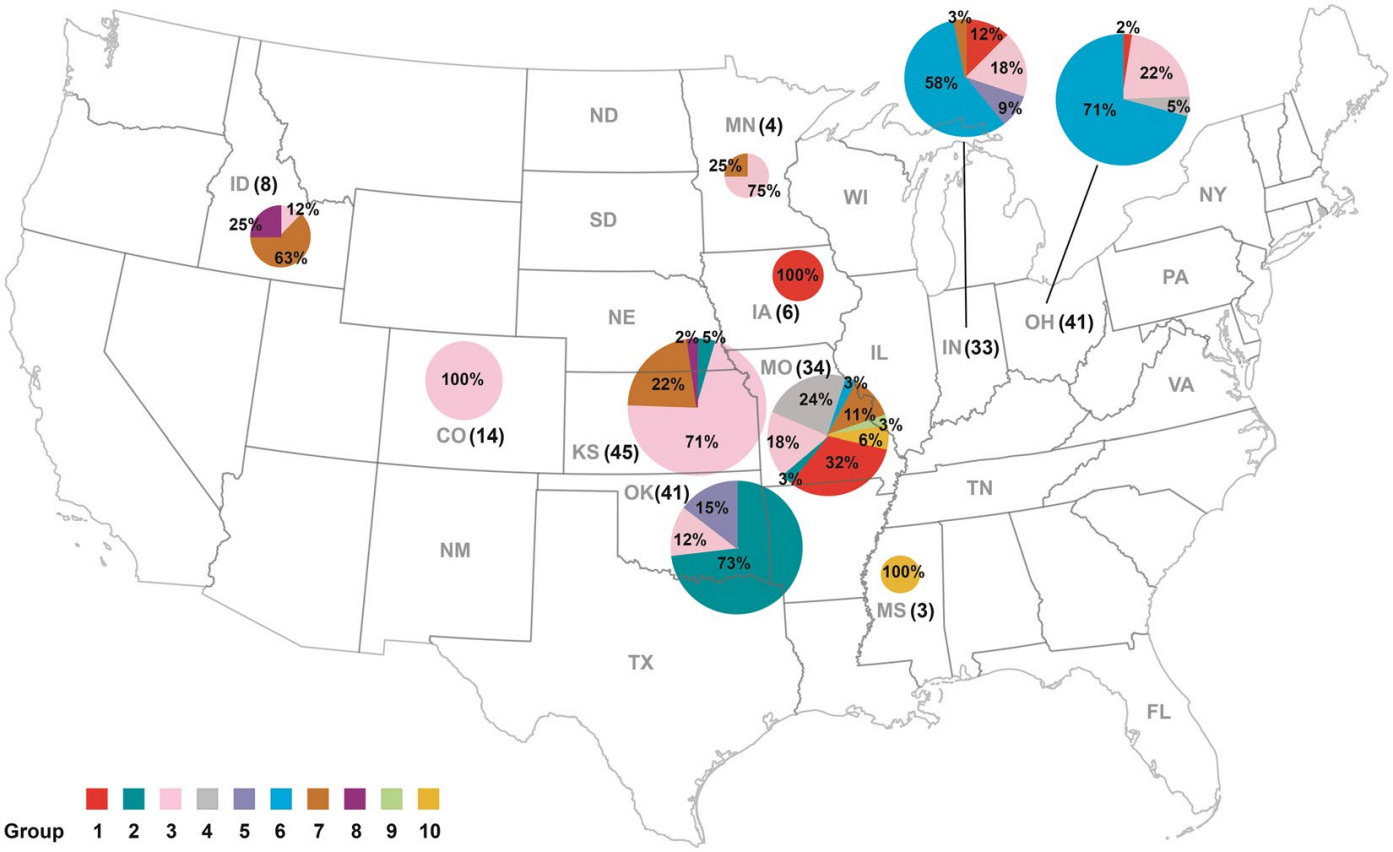
Distribution of *B*. *canis* genotype groups in different states. Number in brackets after the state abbreviation indicate total positives in that state; pie chart indicates percentages of each genotype group in that state. Pie size corresponds to the number of isolates. Those with state abbreviations but without a number indicate samples that were collected but all tested negative (number of submission are shown in Table 1). No sample was collected from those that do not have the state abbreviation. Different colors indicate different genotype groups. Red: genotype group 1; dark teal: group 2; pink: group 3; gray: group 4; lavender: group 5; light teal: group 6; orange: group 7; maroon: group 8; light green: group 9; and light orange: group 10. The map template was downloaded from www.vectortemplates.com (Graphics Factory CC, Western Cape, Sourth Africa) with permission.

A cluster analysis of the MLVA allele profiles of the 229 *B*. *canis* isolates with nine reference sequences by BioNumerics generated three large clusters, Cluster A, Cluster B and Cluster C, as well as 3 sub-clusters, C1, C2 and C3 (Fig. 1). Distribution the 229 isolates in different states is shown in Fig. 2. Cluster A mainly contained isolates from Oklahoma in 2010 (8/9) and 2011 (22/32), as well as all the isolates from Iowa in 2013 (6/6). Cluster B mainly consisted of isolates from Indiana (19/25) and Ohio in 2013 (18/33), as well as from Ohio in 2012 (6/8). Most isolates in Cluster C were from Kansas (43/45) and Idaho (7/8). All isolates from Colorado (14/14) and Missouri (3/3) were also classified into Cluster C. Isolates from Indiana in 2013 were in Cluster B, while isolates in other years (2012, 2015 and 2016) were all in Cluster C. Isolates in different years were spread in different clusters except samples collected in 2015 and 2016 (Fig. 1). Most isolates in group 1 (81.8%, 18/22) and 2 (94.1%, 32/34) belonged to Cluster A, and most isolates in group 6 (83.6%, 41/49) belonged to Cluster B. All isolates in group 4 (10), 7 (21) and 10 (5) as well as most isolates in group 3 (64/76) and 5 (8/9) belonged to Cluster C. The five isolates each in groups 8 and 9 were located in Cluster A and C, respectively (Insert in Fig. 1).

### Distributions of *B*. *canis* isolates in different dog breeds and genders

Breed information was available for 118 *B*. *canis*-positive dogs, which included 24 different dog breeds. Isolates from German Shepherd, French Bulldog, Siberian Husky and Bichon Frise were mostly grouped in Cluster A. All four isolates from Cocker Spaniel and five out of 20 isolates from Shih Tzu were in Cluster B. Isolates collected from more breeds were in Cluster C (Supplementary Table S1). These Cluster C isolates include 15 of 20 isolates from Shih Tzu, 11 of 12 from Poodle, 8 of 9 from Pomeranian, all 6, 5, and 4 isolates from Miniature Schnauzer, Chihuahua and Golden Retriever, respectively, and a few each from other breeds. Gender information was available for 133 positive dogs (Supplementary Table S1) including 87 females and 46 males. No correlation between animal gender and *B*. *canis* genotype was observed.

### Stability of the 13 VNTR loci on *B*. *canis* genomes

**Figure 3:**
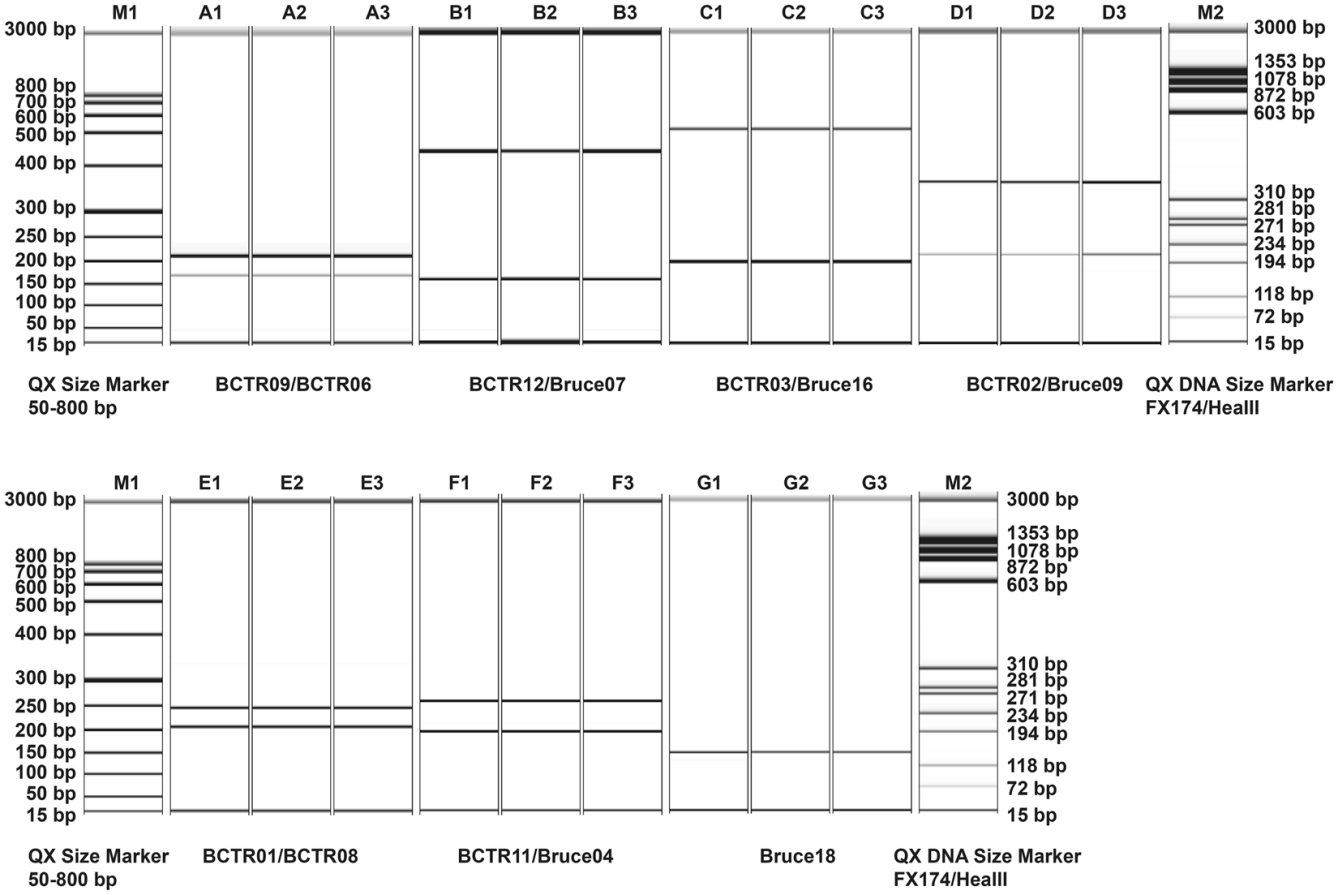
Electrophoresis images of *B*. *canis* isolate D13-123417 #42 amplified with 13 VNTR locus primers after *in vitro* propagations. Lane A1–A3: original, 10^th^ and 20^th^ subcultures by BCTR09 (upper) and BCTR06 (lower); Lane B1–B3: original, 10^th^ and 20^th^ subcultures by BCTR12 (upper) and Bruce07 (lower); Lane C1–C3: original, 10^th^ and 20^th^ subcultures by BCTR03 (upper) and Bruce16 (lower); Lane D1–D3: original, 10^th^ and 20^th^ subcultures by BCTR02 (upper) and Bruce09 (lower); Lane E1–E3: original, 10^th^ and 20^th^ subcultures by BCTR01 (upper) and BCTR08 (lower); Lane F1–F3: original, 10^th^ and 20^th^ subcultures by BCTR11 (upper) and Bruce04 (lower); Lane G1–G3: original, 10^th^ and 20^th^ subcultures by Bruce18. Lane M1: QX Size Marker 50–800 bp. Lane M2: QX Size Marker FX174/HeaIII.

The original isolates from samples D11-106637 #69 that were collected in 2011, D13-123417 #42 in 2013, and D16-008400 #3 in 2016, and their 10^th^ and 20^th^ subcultures were used for DNA extractions and MLVA-13Bc PCR. For each isolate, identical MLVA-13Bc profiles were generated for the original isolates, and their 10^th^ and 20^th^ subcultures, indicating the 13 VNTRs were stable during propagation. The data of 13 VNTR loci on the original isolate, its 10^th^ and 20^th^ subcultures of sample D13-123417 #42 is shown in Fig. 3.

## Discussion

A highly discriminatory MLVA assay was developed and validated with a relatively large number of *B*. *canis* isolates. With the combination of the 13 VNTRs, each of the 229 isolates were differentiated. This MLVA-13Bc assay was specifically designed for genotyping of *B*. *canis* strains with high discriminatory power. The MLVA-13Bc assay established and validated in this study can be a useful tool in epidemiological studies, especially during *B*. *canis* outbreaks.

The MLVA-16 assay^24^ is probably the most used method for genotyping different *Brucella* spp., and for strains within certain *Brucella* spp. including *B*. *canis*. As the method was not designed specifically to differentiate strains within *B*. *canis*, it provided limited discriminatory power against *B*. *canis* strains. For example, MLVA-16 differentiated the 29 Chinese *B*. *canis* strains into 21 genotypes, as reported by Di *et al*.^25^. Kang *et al*.^29^ classified 77 Korean *B*. *canis* strains into 30 genotypes using the same procedure. As we are using a different procedure, those strains could not be *in silico* analyzed except for the nine reference strains that have full or near full genome sequences. Our MLVA-13Bc assay differentiated each of the 229 strains used in this study. In comparison, the five most informative loci from the MLVA-16 assay classified the 229 strains into 131 genotypes. To further confirm that the remaining 11 VNTRs in the MLVA-16 assay would not generate useful information for *B*. *canis* strains, we have tested the 11 primer pairs on each of the genotype groups, and resulted identical amplicon sizes (Figure S3). Our data from this study together with other published data suggested that the remaining 11 VNTRs from the MLVA-16 assay do not provide useful discriminatory power for *B*. *canis* genotyping.

A survey on canine infection with *B*. *canis* was carried out in 22 U.S. states between 2010 and 2016, and the result indicated that the total average positive rate is 5.5% (377/6,844; Table 1). Although *B*. *canis* can cause human infections, information of the zoonotic potential of these 229 strains are not available. Due to not all positive samples in years 2010 and 2015, and none in 2014 were subjected to bacterial isolation, the more realistic *B*. *canis* isolation rate is reflected by data in years 2011, 2012, 2013 and 2016, which was 79.5% (194/244). This data demonstrated a good correlation between the culture and the molecular detection methods, yet about 20% of PCR positives would not be detected by the traditional culture method, indicating PCR can be more sensitive in *B*. *canis* detection.

Dog breed information was available for about half of our strain collection. We identified 24 different dog breeds from 118 out of 229 *B*. *canis* isolates. Although we did not see a clear-cut distribution, isolates from certain dog breeds were primarily located in a given cluster (Table S1). Clusters A and B are smaller clusters. Isolates from German Shepherd, French Bulldog, Siberian Husky and Bichon Frise were mostly grouped in Cluster A, and all four isolates from Cocker Spaniel and five out of 20 isolates from Shih Tzu were in Cluster B. We also have dog age information for 60 samples, but there was no correlation between dog age and isolate genotypes observed (Table S1).

There were 133 positive samples (Supplementary Table S1) that were indicated with animal genders. Although we received more samples from females (n = 87) than males (n = 46), it is worth mentioning that we received a significant portion from *B*. *canis*-barring male animals that has not been extensively documented, as most of the reports were on abortion cases^9, 30^. Our data indicated that the organism can circulate in blood streams of both male and female animals. Thus for *B*. *canis* screening, especially for breeding facilities, it is important to screen both female and male breeding dogs.

Our MLVA genotyping data on 229 *B*. *canis* strains may be the largest genotyping study for *B*. *canis* isolates in the US. Although the distribution of different genotypes in each state was not clear-cut, some interesting information was revealed. Genotype group 6 was the predominant genotype for both Indiana and Ohio, which are neighbor states. Group 1 isolates were mainly distributed in Missouri, Iowa and Indiana, states that are geographically related. Group 2 isolates were mainly found in Oklahoma and some in Missouri, which are also bordering states. Groups 3 and 7 though were widely distributed in different states.

In a temporal distribution analysis for these 229 *B*. *canis* isolates, all 18 samples collected in 2016 were in Cluster C. Most isolates in 2015 (12/13) were in Cluster C, only one was in Cluster A. Most isolates collected from 2011 (28/38) were in Cluster A, some (9/38) were in Cluster C and only one in Cluster B.

In conclusion, most currently-used MLVA assays (MLVA-8, MLVA-10, MLVA-11, MLVA-15 and MLVA-16) were not specifically designed for *B*. *canis* at the strain level, although they are good molecular genotyping methods of *Brucella* at the species, biovar or at strain level for certain *Brucella* species. Our newly developed MLVA-13Bc method was specifically designed to discriminate strains from within the species of *B*. *canis*, and had higher discriminatory power than previous assays^25, 31, 32^. Although our data is not associated with pathogenesis as such information is not available, the new MLVA-13Bc method genotyped each of the 229 isolates in our collection, indicating that it is a useful molecular epidemiological tool to trace the source of strain introduction in an event of a *B*. *canis* outbreak.

## Methods

### *B*. *canis* sample preparation

From January 2010 to May 2016, canine whole blood samples (n = 6,844) from 22 states were submitted to the Kansas State Veterinary Diagnostic Laboratory (KSVDL) for *B*. *canis* identifications (Table 1). All samples were diagnostic samples submitted by clients and no experimental samples were used. All procedures were carried out strictly following institutional biosafety procedures. Samples were freshly collected in sodium citrate (blue-top) or EDTA (purple-top) blood collection tubes, and delivered to KSVDL on ice for next-day delivery. Upon receiving, samples were accessioned and 1 ml blood was transferred into 5 mL Brain Heart Infusion (BHI) broth (HARDY Diagnostics, Santa Maria, CA, USA), mixed, and kept at −80 °C for 3 h (for samples received in the mornings) or at −20 °C for overnight (for those received in the afternoons), then incubated at 37 °C aerobically in a shaker incubator for 40–48 h prior to DNA extraction. A duplex PCR assay was used for *B*. *canis* identification. Isolates used in this study were confirmed by culture isolation followed by PCR confirmation on single colonies, and by sequencing on selected isolates (detailed below).

### DNA extraction, PCR identification and sequencing confirmation

After the 40–48 h incubation, 150 µL of culture was used for DNA extraction with the DNeasy Blood & Tissue Kit (Qiagen, Valencia, CA, USA) following the manufacturer’s protocol. Extracted DNA was subjected to a duplex PCR assay that was composed of two molecular targets: a 308 bp fragment of the rRNA gene that is present in all *Brucella* spp. (forward primer: BruCom-F4, CCGCCTTCGTTTCTCTTTCT; reverse primer: BruCom-R4, GGGATCGAACCGACGAC); and a 185 bp *B*. *canis*-specific region (1,161 bp for non-*canis Brucella* spp.) flanking the 976 bp deletion^27^ that occurs only in *B*. *canis* strains (forward primer: BcanisF, GGCTGTCAAGGCGATAAAAC; reverse primer: BcanisR, CAGCTTTACTGCCGGGTTAG). The 20 µl PCR reaction contained 1 µl of the primer mix (0.4 µM for each primer), 10 µL of BioRad (Hercules, CA) iQ Multiplex Powermix, 2 µL of extracted DNA, and 7 µL nuclease-free water. The PCR amplification program included a 5 min denaturation at 95 °C, followed by 35 cycles of 95 °C for 20 sec, and 60 °C for 50 sec. The PCR products were separated and visualized on a Qiagen QIAxcel capillary electrophoresis system. The 185 bp PCR product from selected isolates was outsource sequenced for confirmation.

### Isolation of *B*. *canis* from PCR positive samples

Culture isolation of *B*. *canis* was achieved by streaking the BHI culture on 5% blood agar plates (HARDY Diagnostics). The inoculated plates were incubated at 37 °C aerobically for 72–120 h. Single colonies morphologically consistent with *Brucella* were sub-cultured in BHI broth for 24 h, and confirmed by PCR. All isolates confirmed as *B*. *canis* were stored with 15% glycerol at −80 °C for long-term use.

### Development of the Multiple Locus Variable-Number Tandem Repeat Analysis method for *B*. *canis* genotyping

Published data using MLVA-15^22^ or MLVA-16^24, 29^ methods were analyzed with an emphasis on their ability to differentiate *B*. *canis* strains. The purpose of analysis on published data was to select those VNTRs that generated polymorphism on *B*. *canis* strains and to compile them with new VNTRs (described below) to form a new MLVA method that can be used for genotyping of *B canis* strains with increased discriminatory power. An effort of identifying additional VNTR loci was performed by searching *B*. *canis* whole genome sequences (chromosome I: NC_010103, NC_016778, NZ_CP007758, NZ_CP007629; chromosome II: NC_010104, NC_016796, NZ_CP007759, NZ_CP007630) using Tandem Repeats Finder described by Benson^33^. Additional VNTR loci were selected based on following criteria recommended by Nadon *et al*.^34^: all loci had no insertions and deletions in the tandem repeats; each tandem repeat was equal or greater than 8 bp; and the loci had conserved flanking sequences from which the PCR primers can be identified. Primers flanking each locus were designed using the online primer design software, Primer 3 (http://bioinfo.ut.ee/primer3-0.4.0/). Twenty *B*. *canis* isolates selected from different dog breeds and different colleting states spanning different years were used to evaluate and optimize the method. All individual VNTR were run on the 20 isolates first, and the optimized protocol was used for the remaining isolates.

### Multiple Locus Variable-Number Tandem Repeat Analysis procedure

*B*. *canis* stocks stored at −80 °C were streaked on blood agar plates, and cultured at 37 °C for 72–120 h. A single colony from each plate was inoculated into 5 ml BHI broth and incubated at 37 °C with shaking for 48–72 h. The resulting culture was used for genomic DNA extraction with Qiagen DNeasy Blood & Tissue Kit. Takara Premix Taq™ DNA Polymerase Hot-Start Version kit (Clontech Laboratories, Mountain View, CA, USA) was used for PCR amplifications. The 20 μL PCR reaction contains 11.9 μL (13.4 μL for Reaction 7) double distilled water, 2 μL 10 × buffer, 1.5 μL each primer pair (final concentration of 375 pM for each primer), 1 μL dNTP Mix (2.5 mM each), 0.1 μL rTaq enzyme, and 2 μL of extracted DNA. The thermal cycling profile consisted of an initial denaturation at 95 °C for 5 min, followed by 35 cycles of 95 °C for 30 sec, 58 °C for Reactions 1, 2 and 3, 60 °C for Reactions 4 and 7, or 62 °C for Reactions 5 and 6 for 30 sec and 72 °C for 45 sec, plus a final extension step at 72 °C for 5 min. VNTR PCR amplicons were separated and visualized on a Qiagen QIAxcel Advanced System with QX DNA Size Marker FX174/III and QX Size Marker 50–800 bp. Fragment analysis was performed with QIAxcel Biocalculator software. Selected amplicons of different sizes obtained by QIAxcel Advanced System were verified by Sanger DNA sequencing.

### MLVA data analysis

The genotype of each isolate was identified by compiling the genotype generated by each VNTR locus. The phylogenetic tree was generated using an unweighted pair group method with arithmetic mean (UPGMA) method^35^ that is embedded in BioNumerics version 7.6 (Applied Maths, Austin, TX). The cluster analysis was performed using the UPGMA with a minimum spanning tree (MST) and distance matrices for categorical data by BioNumerics version 7.6. The Geographic distribution map template was downloaded from www.vectortemplates.com (Graphics Factory CC, Western Cape, Sourth Africa) with permission, and the added pie charts were constructed using BioNumerics version 7.6. Hunter and Gaston discrimination index (HGDI) was calculated using Ridom EpiCompare software version 1.0 (www.ridom.de/epicompare/) to elucidate the discriminatory power of the genotyping methods, which explained the probability of two unrelated and different isolates sampled from the test population grouping as different subtypes by a specific typing method^28^. The 95% confidence intervals (CI) were calculated according to the method previously described^36^.

### VNTR loci stability of the *B*. *canis* genome

In order to investigate the stability of the 13 VNTR loci in the *B*. *canis* genome, three *B*. *canis* isolates collected from 2011 (D11-106637 #69), 2013 (D13-123417 #42), and 2016 (D16-008400 #3) were subcultured by inoculating 50 μL of stock culture into 5 ml BHI medium and incubated at 37 °C for 2 days. A new 5 ml BHI broth was inoculated with 50 μL of fresh culture, and incubated at 37 °C for 2 days. A total of 20 consecutive subcultures were made for each strain. Portions of each subculture were frozen at −20 °C till use. When 20 subcultures were completed, the original culture, subculture number 10 and number 20 were used for DNA extraction and PCR amplification with the newly developed genotyping method.

## Electronic supplementary material

Supplementary Information
