# Supplementary material for: Genotyping Brucella canis isolates using a highly discriminatory multilocus variable-number tandem-repeat analysis (MLVA) assay
Source: Sci Rep. 2017 Apr 21;7:1067. doi: 10.1038/s41598-017-01114-7 (PMC5430899; doi:10.1038/s41598-017-01114-7)
Supplement: Supplementary file 1 — Supplementary Information [file 41598_2017_1114_MOESM1_ESM.doc]

**Supplementary Dataset**

**Genotyping *Brucella canis* isolates using a highly discriminatory multilocus variable-number tandem-repeat analysis (MLVA) assay**

Yi Yang, Yin Wang, Elizabeth Poulsen, Russell Ransburgh, Xuming Liu, Baoyan An, Nanyan Lu, Gary Anderson, Chengming Wang and Jianfa Bai

**Supplementary Table S1. Distribution of different clusters and genotype groups with host dog breeds, gender, and age information**

| **Dog breed** | **Cluster-A** | **Cluster-B** | **Cluster-C** | **Total** | **G1**^a^ | **G2** | **G3** | **G4** | **G5** | **G6** | **G7** | **G8** | **G9** | **G10** |
| --- | --- | --- | --- | --- | --- | --- | --- | --- | --- | --- | --- | --- | --- | --- |
| Bichon Frise | 3 | 1 | 2 | **6** | 0 | 0 | 4 | 0 | 0 | 1 | 0 | 1 | 0 | 0 |
| Boston Terrier | 0 | 0 | 1 | **1** | 0 | 0 | 0 | 0 | 0 | 0 | 0 | 1 | 0 | 0 |
| Cavalier K.C. Spaniel | 0 | 0 | 3 | **3** | 0 | 0 | 2 | 0 | 0 | 0 | 1 | 0 | 0 | 0 |
| Chihuahua | 0 | 0 | 5 | **5** | 0 | 0 | 4 | 0 | 0 | 0 | 0 | 0 | 0 | 1 |
| Cocker Spaniel | 0 | 4 | 0 | **4** | 0 | 0 | 0 | 0 | 0 | 4 | 0 | 0 | 0 | 0 |
| Dachshund | 1 | 0 | 4 | **5** | 0 | 0 | 5 | 0 | 0 | 0 | 0 | 0 | 0 | 0 |
| French Bulldog | 6 | 0 | 1 | **7** | 6 | 0 | 0 | 0 | 0 | 0 | 1 | 0 | 0 | 0 |
| German Shepherd | 6 | 0 | 0 | **6** | 6 | 0 | 0 | 0 | 0 | 0 | 0 | 0 | 0 | 0 |
| Golden Retriever | 0 | 0 | 4 | **4** | 0 | 0 | 0 | 1 | 0 | 0 | 1 | 0 | 0 | 2 |
| Siberian Husky | 4 | 0 | 0 | **4** | 4 | 0 | 0 | 0 | 0 | 0 | 0 | 0 | 0 | 0 |
| Italian Greyhound | 0 | 0 | 1 | **1** | 0 | 0 | 1 | 0 | 0 | 0 | 0 | 0 | 0 | 0 |
| Maltese | 0 | 0 | 3 | **3** | 0 | 0 | 3 | 0 | 0 | 0 | 0 | 0 | 0 | 0 |
| Miniature Pinscher | 0 | 0 | 2 | **2** | 0 | 0 | 1 | 0 | 0 | 0 | 1 | 0 | 0 | 0 |
| Miniature Schnauzer | 0 | 0 | 6 | **6** | 0 | 0 | 6 | 0 | 0 | 0 | 0 | 0 | 0 | 0 |
| Mixed breed | 0 | 0 | 1 | **1** | 0 | 0 | 1 | 0 | 0 | 0 | 0 | 0 | 0 | 0 |
| Papillon | 0 | 0 | 1 | **1** | 0 | 0 | 1 | 0 | 0 | 0 | 0 | 0 | 0 | 0 |
| Pomeranian | 1 | 0 | 8 | **9** | 0 | 1 | 6 | 0 | 0 | 0 | 0 | 0 | 0 | 2 |
| Poodle | 1 | 0 | 11 | **12** | 0 | 1 | 4 | 1 | 1 | 1 | 3 | 1 | 0 | 0 |
| Pug | 0 | 0 | 1 | **1** | 0 | 0 | 0 | 0 | 0 | 0 | 0 | 0 | 1 | 0 |
| Schnauzer | 0 | 1 | 3 | **4** | 0 | 0 | 1 | 0 | 0 | 0 | 3 | 0 | 0 | 0 |
| Shiba Inu | 0 | 0 | 1 | **1** | 0 | 0 | 0 | 1 | 0 | 0 | 0 | 0 | 0 | 0 |
| Shih Tzu | 0 | 5 | 15 | **20** | 0 | 0 | 12 | 0 | 0 | 5 | 3 | 0 | 0 | 0 |
| Australian Silky | 0 | 0 | 2 | **2** | 0 | 0 | 2 | 0 | 0 | 0 | 0 | 0 | 0 | 0 |
| Vizsla | 0 | 0 | 2 | **2** | 0 | 0 | 2 | 0 | 0 | 0 | 0 | 0 | 0 | 0 |
| Yorkshire Terrier | 1 | 0 | 7 | **8** | 1 | 0 | 0 | 0 | 2 | 0 | 5 | 0 | 0 | 0 |
| Total | 23 | 11 | 84 | **118** | 17 | 2 | 55 | 3 | 3 | 11 | 18 | 3 | 1 | 5 |
| **Gender** | **Cluster-A** | **Cluster-B** | **Cluster-C** | **Total** | **G1** | **G2** | **G3** | **G4** | **G5** | **G6** | **G7** | **G8** | **G9** | **G10** |
| Male | 7 | 7 | 32 | **46** | 6 | 0 | 18 | 1 | 1 | 8 | 7 | 1 | 1 | 2 |
| Female | 17 | 10 | 60 | **87** | 13 | 2 | 43 | 3 | 2 | 10 | 10 | 1 | 0 | 3 |
| Total | 24 | 17 | 92 | **133** | 19 | 2 | 61 | 4 | 3 | 18 | 17 | 2 | 1 | 5 |
| **Age** | **Cluster-A** | **Cluster-B** | **Cluster-C** | **Total** | **G1** | **G2** | **G3** | **G4** | **G5** | **G6** | **G7** | **G8** | **G9** | **G10** |
| 1 | 2 | 1 | 5 | **8** | 2 | 0 | 0 | 0 | 2 | 1 | 1 | 0 | 0 | 2 |
| 2 | 6 | 0 | 13 | **19** | 3 | 0 | 6 | 1 | 0 | 0 | 4 | 2 | 1 | 2 |
| 3 | 8 | 2 | 6 | **16** | 8 | 0 | 3 | 1 | 1 | 3 | 0 | 0 | 0 | 0 |
| 4 | 1 | 3 | 4 | **8** | 1 | 0 | 2 | 1 | 0 | 3 | 0 | 0 | 0 | 1 |
| 5 | 1 | 1 | 1 | **3** | 1 | 0 | 1 | 0 | 0 | 1 | 0 | 0 | 0 | 0 |
| 6 | 2 | 0 | 2 | **4** | 0 | 0 | 3 | 0 | 0 | 0 | 0 | 1 | 0 | 0 |
| 7 | 1 | 1 | 0 | **2** | 0 | 1 | 0 | 0 | 0 | 1 | 0 | 0 | 0 | 0 |
| Total | 21 | 8 | 31 | **60** | 15 | 1 | 15 | 3 | 3 | 9 | 5 | 3 | 1 | 5 |

^a^ G: Genotype group

**
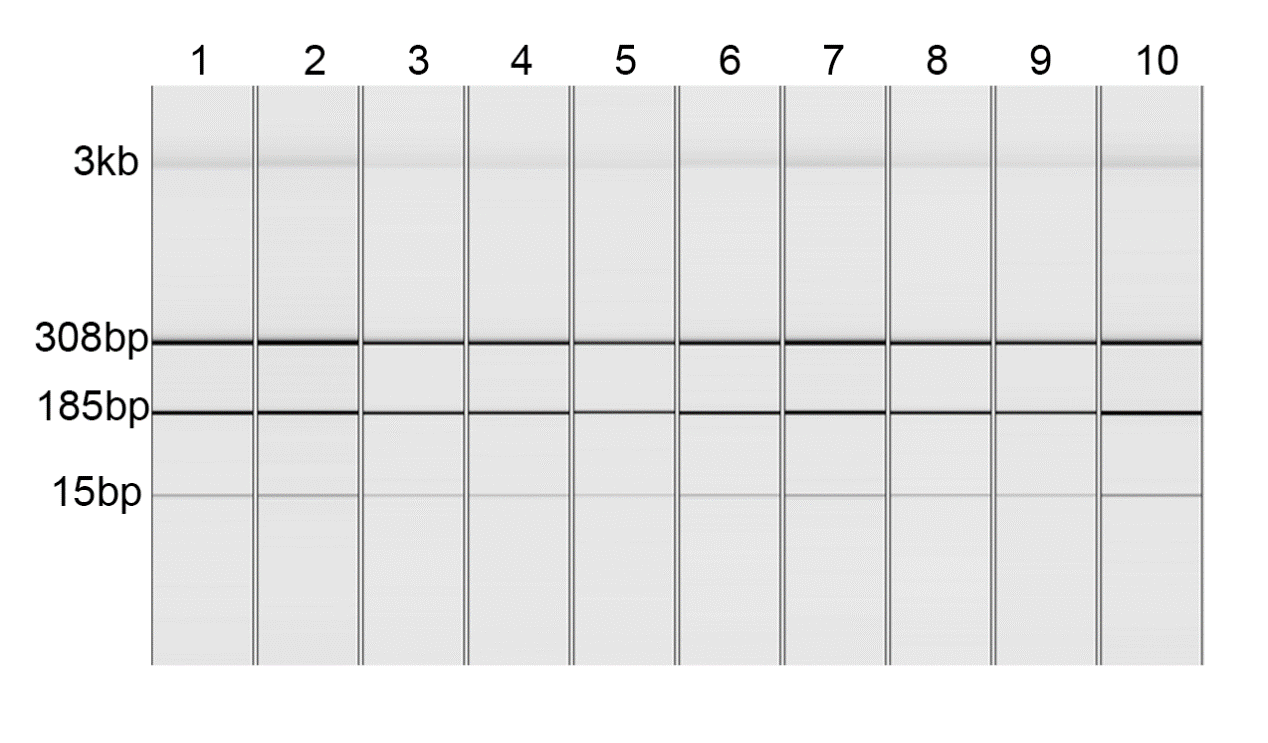
**

**Supplementary Figure S1. PCR amplification on *B. canis* genotype groups 1-10 as indicated by the number on top of each lane.** The PCR products were separated and visualized by QIAxcel. The 3 kb and 15 bp are QIAxcel markers; the 308 bp was a fragment of the rRNA gene present in all *Brucella* species; the 185 bp was a specific amplicon for *B. canis* only.

**
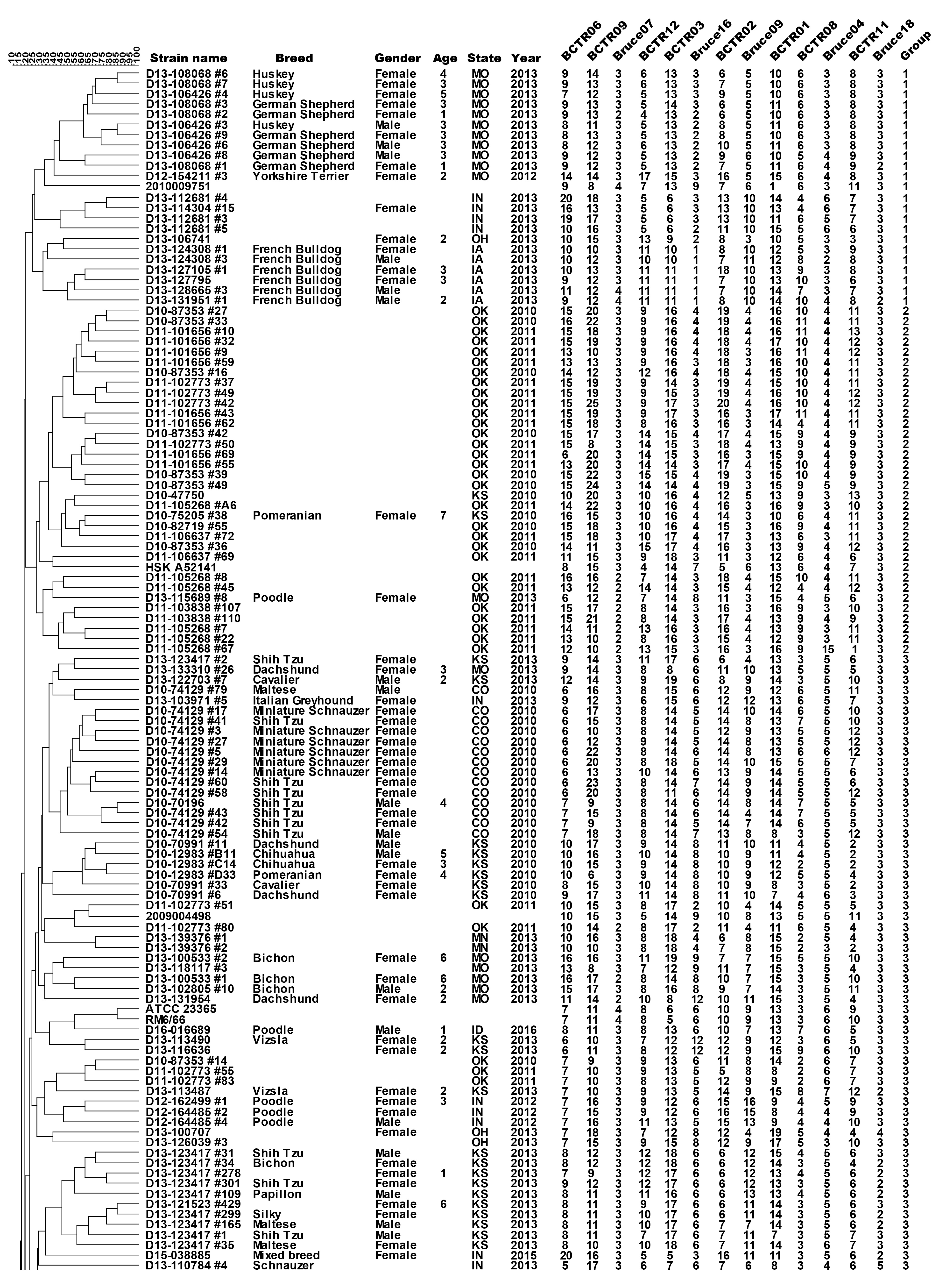
**

**
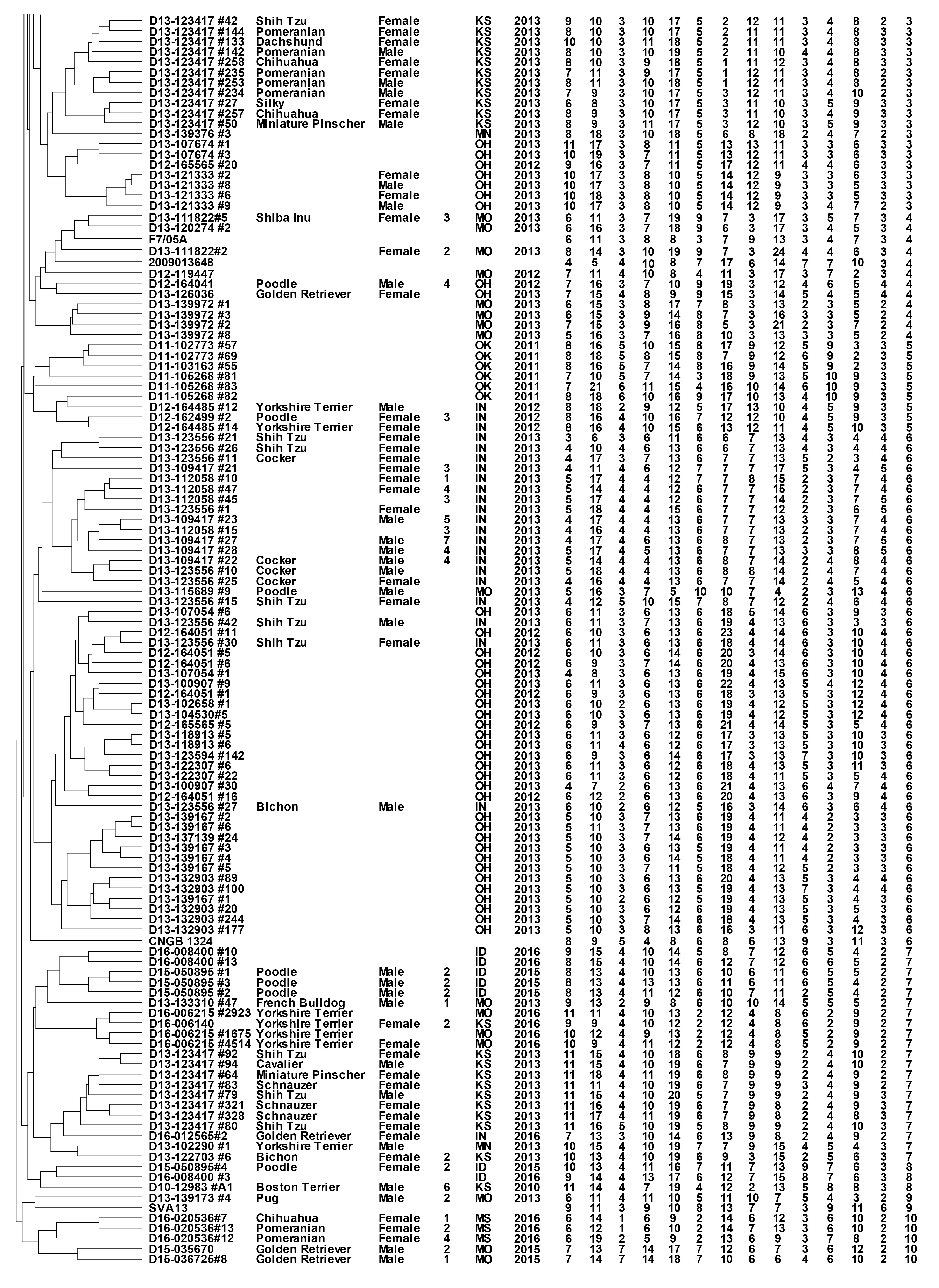
**

**Supplementary Figure S2. Sample information and genotypes of 229 *B. canis* isolates analyzed by the newly developed MLVA-13Bc assay.** From left to right, the columns designate strain name, host dog breed, gender, age, collecting state, sampling year, repeat number of 13 loci (BCTR06, BCTR09, bruce07, BCTR12, bruce16, BCTR03, bruce09, BCTR02, bruce18, BCTR08, BCTR01, bruce04 and BCTR11), and genotype group.


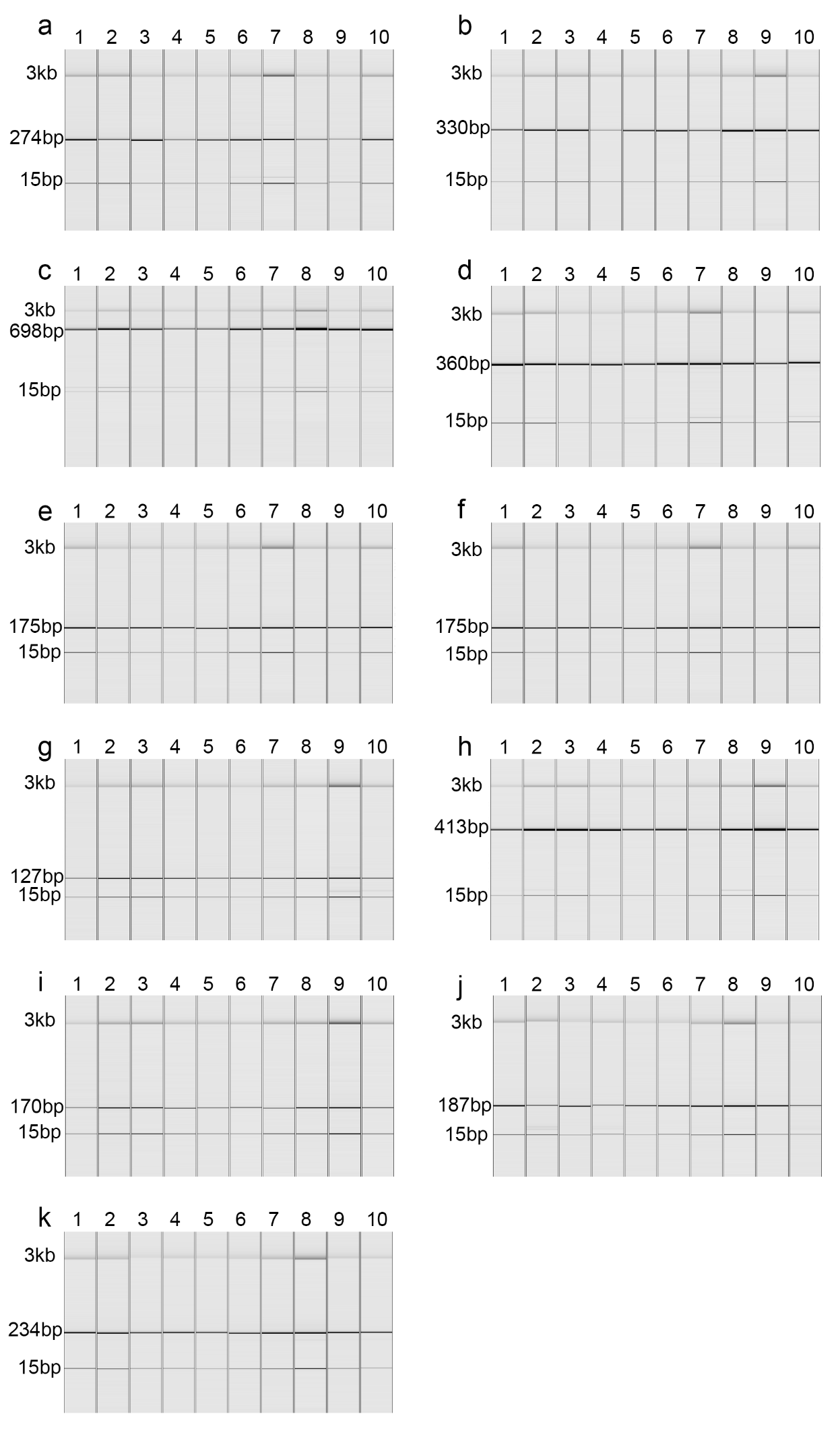


**Supplementary Figure S3. Amplicons generated by the 11 VNTRs from the MLVA-16 method that were not used in MLVA-13Bc assay**. Strains were selected to represent each genotype groups as indicated by numbers on top of each lane. Panels *a-k* represent loci Bruce06, Bruce08, Bruce11, Bruce12, Bruce42, Bruce43, Bruce45, Bruce55, Bruce18, Bruce19, and Bruce30, respectively.
